# Morphohistological Features of Pancreatic Stump Are the Main Determinant of Pancreatic Fistula after Pancreatoduodenectomy

**DOI:** 10.1155/2014/641239

**Published:** 2014-05-13

**Authors:** Cristina Ridolfi, Maria Rachele Angiolini, Francesca Gavazzi, Paola Spaggiari, Maria Carla Tinti, Fara Uccelli, Marco Madonini, Marco Montorsi, Alessandro Zerbi

**Affiliations:** ^1^Section of Pancreatic Surgery, Department of General Surgery, Humanitas Research Hospital, University of Milan School of Medicine, Via Manzoni 56, Rozzano, 20089 Milan, Italy; ^2^Department of Pathology, Humanitas Research Hospital, Rozzano, 20089 Milan, Italy

## Abstract

*Introduction.* Pancreatic surgery is challenging and associated with high morbidity, mainly represented by postoperative pancreatic fistula (POPF) and its further consequences. Identification of risk factors for POPF is essential for proper postoperative management. *Aim of the Study.* Evaluation of the role of morphological and histological features of pancreatic stump, other than main pancreatic duct diameter and glandular texture, in POPF occurrence after pancreaticoduodenectomy. *Patients and Methods.* Between March 2011 and April 2013, we performed 145 consecutive pancreaticoduodenectomies. We intraoperatively recorded morphological features of pancreatic stump and collected data about postoperative morbidity. Our dedicated pathologist designed a score to quantify fibrosis and inflammation of pancreatic tissue. *Results.* Overall morbidity was 59,3%. Mortality was 4,1%. POPF rate was 28,3%, while clinically significant POPF were 15,8%. Male sex (*P* = 0.009), BMI ≥ 25 (*P* = 0.002), prolonged surgery (*P* = 0.001), soft pancreatic texture (*P* < 0.001), small pancreatic duct (*P* < 0.001), pancreatic duct decentralization on stump anteroposterior axis, especially if close to the posterior margin (*P* = 0.031), large stump area (*P* = 0.001), and extended stump mobilization (*P* = 0.001) were related to higher POPF rate. Our fibrosis-and-inflammation score is strongly associated with POPF (*P* = 0.001). *Discussion and Conclusions.* Pancreatic stump features evaluation, including histology, can help the surgeon in fitting postoperative management to patient individual risk after pancreaticoduodenectomy.

## 1. Introduction

Pancreatoduodenectomy (PD) has become over the years the treatment of choice for benign and malignant diseases of the periampullary region [1, 2]. The outcomes of this procedure gradually improved, due to more accurate indications and advances in surgical techniques and in perioperative care (before, during, and after surgery) and also by pancreatic surgery centralization in high-volume centers [3]. All these improvements have led to a considerable and progressive decrease in mortality, keeping rates below 5% in referral centers; nevertheless, similar effects are still not observed on morbidity, remaining close to 50%, even in high-volume surgical settings as described by large series in the literature [4].

Postoperative pancreatic fistula (POPF) is frequently observed, with reported incidence between 8% and 30%, and substantially contributes to overall morbidity [5]. This complication can have catastrophic consequences, particularly sepsis and hemorrhage, and remains the leading risk factor for postoperative death, longer hospital stay, and increased hospital costs after PD [6–8]. For these reasons, a reliable POPF prediction could lead to postoperative management fitting to patient personal risk.

In recent years there has been considerable interest in POPF risk factors, searching for strategies for its prevention; the choice of technical tricks and the improvement of patient perioperative management were the most debated. Several risk factors for POPF were proposed in the literature: amongst them, most authors focused on soft pancreas, pancreatic duct caliber, the underlying pancreatic pathology, regional blood supply, and surgeon's experience [9–13].

In detail, macroscopically normal or soft pancreas, especially in presence of a small pancreatic duct, sets up a more technically challenging anastomosis that is ultimately more prone to develop a postoperative leakage. Different studies examined morphological features of the pancreatic stump, searching for their relationship with POPF onset: they especially analyzed the glandular texture, as intraoperatively assessed by the surgeon, the presence of pancreatic tissue alteration at histology, and main pancreatic duct diameter. However, there are limited reports in the literature describing with statistical significance the association between Wirsung duct diameter, pancreatic texture, pancreatic tissue histology, and POPF occurrence; moreover, there are no studies concerning Wirsung position in pancreatic stump area and its mobilization extent before performing anastomosis.

This study was designed to evaluate the relationship between the development of postoperative pancreatic fistula in patients undergoing PD and intraoperative findings as glandular consistency, main pancreatic duct diameter, and its location in the area of pancreatic stump, in association with histological fibrosis and inflammation of pancreatic tissue.

## 2. Materials and Methods

We derived all mentioned information from our prospective electronic database, regarding all patients undergoing pancreatic surgery at the Section of Pancreatic Surgery, Department of Surgery, Humanitas Research Hospital of Milan, Italy; data collection received the approval of our hospital ethics committee.

Between March 2011 and April 2013 we performed 145 consecutive PD operations for benign and malignant periampullary disease: pancreatic cancer (53%), periampullary cancer (27%), endocrine tumors (7%), pancreatic cystic lesions (7%), chronic pancreatitis (5%), and other indications for surgery (2%). All operations were performed by head surgeon with 25 years of experience in pancreatic surgery helped by a dedicated surgical team. During the reconstruction phase we generally performed a manual end-to-side pancreatojejunostomy in double layer; in few cases we realized a duct-to-mucosa anastomosis. No pancreaticogastrostomies were performed and no ductal stents were used. At the end of each procedure, two laminar drains were routinely left in place, respectively, ventral and dorsal to the pancreaticojejunostomy, and exteriorized through the left flank.

We prospectively recorded surgeon's judgment about pancreatic texture as soft, medium, or hard by palpation of the pancreatic remnant before reconstruction.

**Figure 1:**
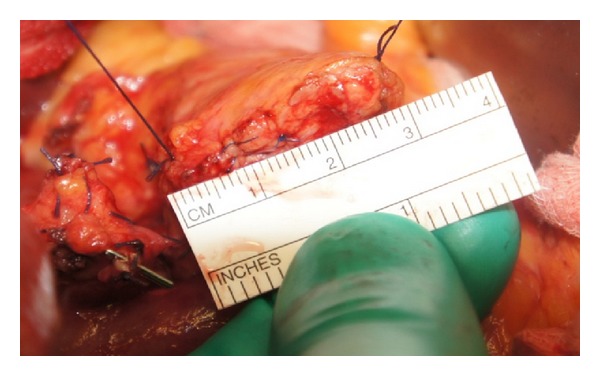
Pancreas measures.

**Figure 2:**
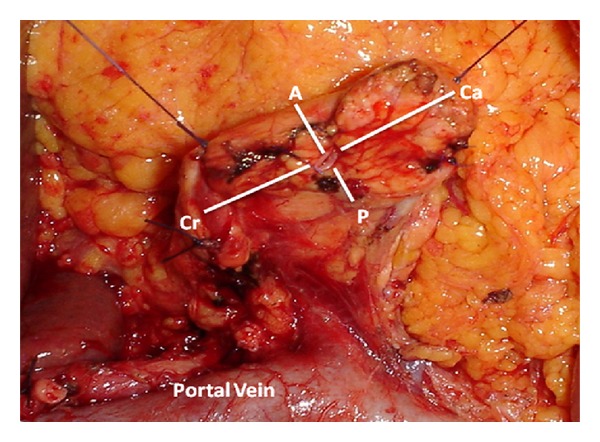
Identification of main pancreatic duct and orthogonal stump axis: craniocaudal (Ca-Cr) and anteroposterior (A-P).

In the intraoperative period, too, we acquired with a sterile ruler the measurement of main pancreatic duct diameter (Figure 1) and of the distance between Wirsung and pancreatic resection margins orthogonally considered (Figure 2) and the extension of gland mobilization from the vessels plane. The whole stump area has been calculated by approximating an ellipse.

An index of Wirsung decentralization has been designed considering the main pancreatic duct position relative to stump orthogonal axes, obtaining a value ranging from −1 to +1 in relation to the area's center: on the craniocaudal axis, the index ranged from −1 to +1 moving from cranial to caudal edge and approaching a zero value near the geometric center of the stump; in a similar way, on the anteroposterior axis, the index ranged from −1 to +1 moving from anterior to posterior margin and reaching a zero value in proximity of the stump center.

We then recorded POPF rate and its clinical impact, according to ISGPF classification [14]. In the postoperative period patients were managed according to our usual clinical protocol: intravenous infusion until the 4th postoperative day, oral feeding from the 4th postoperative day (in absence of clinical contraindications), abdominal CT scan in presence of any clinical or biochemical suspicion of abdominal collection, and specific antibiotic therapy in case of positivity of intraoperative bile culture or of postoperative drain fluid culture. All patients received somatostatin analogues by subcutaneous injection until oral feeding recovery.

### 2.1. Histological Analysis and Score Definition

**Figure 3:**
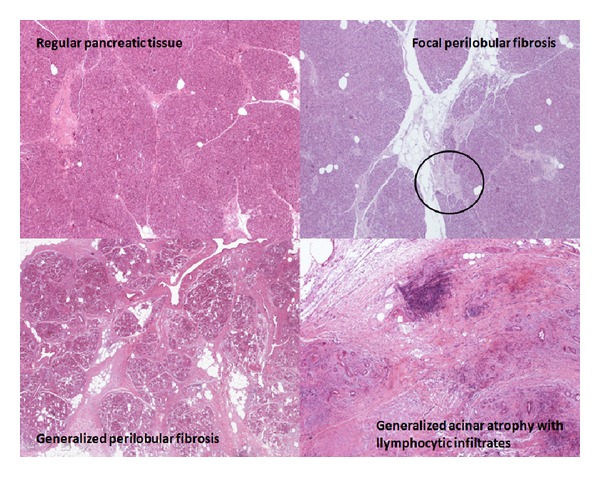
Pancreatic fibrosis and inflammation at histology.

Histological analysis was performed retrospectively by a single dedicate pathologist, who assessed blindly, with optical microscopy, the degree of fibrosis and inflammation of pancreatic tissue on stained slides derived from the resection margin. At pathological analysis the presence of fibrosis was graded on a scale of five levels, starting from normal pancreatic parenchyma, consisting in lobes separated by connective tissue organized in fine septa (“no fibrosis” grade) and reaching the complete replacement of the parenchyma by fibrosis, with rare residual areas of acinar glandular tissue (“subtotal fibrosis” grade). Intermediate steps were identified considering the presence of perilobular fibrosis, (connective tissue involving the lobes, but no penetrating them), focal or extensive, and periacinar fibrosis (fibrosis within the lobes, respecting the acini), focal or extensive, too (Figure 3).

The chronic tissue infiltration by inflammatory lymphocytes was also classified by our pathologist in three grades: absent, focal, or generalized.

A numeric progressive value was given to each grade of fibrosis and each grade of inflammation. As shown in Table 1, values resulting from the sum of the valid points for each criterion were further classified into three groups:

- Group I: normal pancreas or with mild alterations;
- Group II: pancreas with moderate fibrosis and inflammation;
- Group III: pancreas with severe fibrosis and inflammation.

### 2.2. Statistical Analysis

All calculations were realized with PASW Statistics 18th version (SPSS Inc., Chicago, IL). Data were appropriately analyzed by using Student's *t*-test, Wilcoxon test, and chi-square test. A linear regression was employed for multivariate analysis considering significant variables at univariate analysis.

A *P value* less than 0.05 was considered statistically significant.

## 3. Results

We considered in the analysis all the performed consecutive 145 PD operations. All descriptive statistics regarding preoperative, intraoperative, and postoperative variables are shown in Table 2.

The median length of hospital stay (LOS) was 12 days. Overall morbidity was 59,3%. Single postoperative complication rates are listed in Table 2. There were 6 deaths (4,1%): two patients died at postoperative days 3 and 7, due to intestinal ischemia and stroke, respectively, while the others died due to septic complications and sequels secondary to pancreatic fistula occurrence. The incidence of POPF was 28,3%; clinically significant POPF rate (ISGPF grade B-C) was 15.8%.

Analyzing patient personal risk factors for POPF development (Table 3), the univariate analysis showed that male sex (*P* = 0,009), higher BMI (*P* = 0,002), and prolonged surgery duration (*P* = 0,001) were associated with a higher risk of pancreatic fistula.

We then turn to morphological risk factors analysis (Table 4). A soft pancreatic texture resulted strongly associated whit POPF development (73% versus 14%; *P* < 0,001) and whit high grade POPF, as 42% of patient with soft pancreas developed a high grade fistula, compared to 4% of patient with hard or medium texture (*P* < 0,001).

Main pancreatic duct diameter was smaller among patients who developed fistula compared to the others (*P* < 0,001). We identified a main pancreatic duct caliber cut-off for POPF development equal to 3 mm: Wirsung dilatation over 3 mm had a protective role, as 81% of dilated duct did not experience an anastomotic leak (*P* < 0,002). This cut-off was also reliable for high grade POPF: 65% of patients with clinically relevant fistulas carried a Wirsung duct smaller than 3 mm (*P* = 0,022).

Main pancreatic duct decentralization on the stump anteroposterior axis, especially if close to the posterior margin, was related to higher risk to develop pancreatic fistula (*P* = 0,031), while we did not find a similar correlation on the stump craniocaudal axis. This association did not appear when we considered only high grade POPF.

Continuing morphologic analysis, we observed an increased incidence of POPF when resection area was wider (*P* < 0,001); pancreatic stump in high grade POPF group was larger than the others (223,82 versus 149,59; *P* = 0,003). Finally, POPF rate and high grade POPF rate were higher in more mobilized stumps (resp., *P* < 0,001 and *P* = 0,003).

At multivariate analysis, as shown in Table 5, male gender (*P* = 0,043), soft pancreatic texture (*P* = 0,000), and longer stump mobilization (*P* = 0,001) resulted associated with POPF development. When we considered only high grade POPF (ISGPF B-C), soft pancreatic texture was the only independent factor related to pancreatic leakage (*P* = 0,000; 95%CI: 0,221–0,499).

The fibrosis-and-inflammation score computation was realized on a subgroup of 113 patients, containing the first 113 consecutive PD operations. This subgroup revealed uniformity with respect to the entire pool of patients considering all the principle preoperative variables (age, sex, BMI, preoperative albumin, previous diabetes diagnosis, surgery duration, and blood loss); patient in this subgroup experienced a similar POPF rate, too (*P* = 0,870).

As shown in Table 6, the surgeon judgment about pancreatic texture corresponded to the histological grade of fibrosis (*P* > 0,001): among patients with low score (score 0–2), in only 3 cases the surgeon evaluated the pancreas tissue harder than it really was at histology.

We finally observed a strong association between the patient fibrosis-and-inflammation score and POPF occurrence (*P* < 0,001): pancreas with severe fibrosis and inflammation (score ≥ 3) experienced almost zero fistulas, while 90% of patients with lower scores (the clinic “soft pancreas”) developed a pancreatic leakage (Table 7).

## 4. Discussion

Pancreatic fistula is the “Achilles' heel” of pancreatoduodenectomy, as it represents the major cause of morbidity. There is an extensive literature illustrating many predictive factors for POPF development, classified as patient-related, operative, and gland-related factors [15–17].

A reliable POPF risk prediction could be useful to choose the best management for patients undergoing pancreatic resections, including anastomotic techniques or perioperative precautions [18]. In agreement with the literature, in our experience male sex, high body mass index, and prolonged operation time appear to be predisposing to pancreatic fistula, even if only male sex was significant at multivariate analysis.

In our study, focused on pancreatic fistula occurrence in a series of 145 pancreatic head resections, we found a strong association between anastomotic leakage and anatomy of the pancreatic remnant.

As widely reported by previous studies, texture of pancreatic stump and pancreatic duct diameter are often considered risk factors for POPF [19–21]. In 2000 Yeo et al. [20] found that POPF rate was 0% among patients with hardened remaining pancreas and increased to 25% in patients with soft parenchyma. Other investigations confirmed low POPF rates in the presence of firm pancreatic consistency. These findings are similar in our sample, where 66% of patients with soft gland at macroscopic evaluation experienced pancreatic leakage. This result can be easily explained by the technical difficulties of a pancreatoenteric anastomosis in the presence of a soft, friable tissue, which cannot resist the sutures.

Friess et al. [22] demonstrated that increased fibrosis of pancreatic tissue is associated with decreased exocrine activity, resulting in a reduction of the pancreatic juice output. Conversely, all the factors increasing gland fibrosis, like chronic pancreatitis or cancer, had a protective role, allowing for a more secure anastomosis. For the same reason in our sample, too, no patient with hardened pancreatic texture had anastomotic fistula. Our multivariate analysis confirmed that pancreatic texture is an independent predictive factor for pancreatic fistula: it validates the palpatory prediction by the surgeon as reliable.

In the last years the use of the terms “soft/hard” pancreas became popular among the experts; however, it is based only on the intraoperative palpation of the gland. Some efforts have been dedicated to make the macroscopic judgement more objective, as the intraoperative use of a* durometer* for the evaluation of pancreatic hardness [23]. Other studies showed that the subjective surgical assessment was related to the histological grade of fibrosis [24, 25].

We decided to use the pancreatic sample obtained for intraoperative frozen section as an easily available substrate to quantify objectively the fibrosis and inflammation of the parenchyma; we then compared this result with the surgeon's palpatory evaluation; we finally investigated the eventual relationship with POPF development.

As regards histological grade, 65% of patients with low score, carrying a normal or almost normal pancreas, were classified by the experienced surgeon as patients with “soft” pancreas (*P* < 0.001); we also demonstrated that higher scores were associated with very low rate of pancreatic fistula (3 pt versus 56 pt, *P* < 0.001), showing that the more intense the fibrosis and the inflammation were, the more protective the effect on POPF development was.

The diameter of main pancreatic duct is another determinant of anastomotic leakage. Literature widely demonstrated that a duct size smaller than 3 mm increases POPF risk [10, 26]. In our experience, 63.4% of patients with POPF carried a small duct, while 64.4% of patients without POPF had a duct diameter larger than this cut-off. Patients with mean size of 4.29 mm did not experience POPF, while POPF patients had average values of 3.19 mm (*P* < 0.001). Moreover, the 65% of patients with clinically relevant fistula had a small duct (<3 mm), showing that patients with small diameter were also at higher risk of worst fistulas. These findings can be explained considering the fact that a nondilated pancreatic duct can make the duct-to-mucosa anastomosis difficult or even impossible to perform, even in expert hands.

In the present study we analyzed other less discussed morphological features of the pancreatic stump: the area of the section margin, the mobilization of pancreas remnant, and the pancreatic duct position into the cut surface. The evaluation of these variables as predictive factors of POPF has not yet been debated in the literature. Wellner et al. [25] considered the mobilization of the pancreatic remnant among the risk factors but did not show a statistical association.

On the contrary, our data show that an increased mobilization is associated with POPF development. An explanation could be the following: a wide mobilization was performed in high risk situations (soft pancreas with small duct), to facilitate a deep placement of the jejunum loop behind the pancreatic stump, and ultimately to improve anastomotic outcome. However, at multivariate analysis, a wide mobilization proved to be an independent predictor of anastomotic failure: this result could be explained by a relative ischemia at the cut surface caused by vascular discontinuation and also by the intrinsic characteristics of the pancreatic neck, which is a watershed of the glandular blood supply. Strasberg et al. [27] suggested that pancreatic neck has an increased risk of ischemia when divided. On the basis of these data, a wide mobilization of the pancreatic stump (greater than 2.5 cm) is not recommended.

Concerning the stump area, we identify an increased POPF rate in larger pancreatic areas (219 versus 138 mm^2^, *P* < 0.001). This finding was similar among patients who experienced a clinically relevant fistula. This could be a possible explanation: larger cut surfaces have a higher fat infiltration, which is related to parenchyma softness [28]. Moreover, a wide stump area requires a wider opening in the jejunal loop: the greater the opening, the higher the likelihood of a leakage on the enteric side of the anastomosis, making the fistula a pancreatic-enteric fistula, at higher risk of vessel erosion.

As regards the pancreatic duct position along the anteroposterior or craniocaudal axis, the duct decentralization to the posterior margin showed a significant influence on fistula occurrence. According to this evidence we could assume that a central location has a protective role in the pancreaticojejunal anastomosis tightness, probably because a centrally located Wirsung duct makes it easier to place the opening of the jejunal loop accurately in front of the pancreatic stump, performing a more tension-free anastomosis. Moreover, when the duct is close to the posterior margin, less pancreatic parenchyma can be encompassed by stitches placed inside the Wirsung duct, making them at higher risk of failure.

Besides texture and morphological features of pancreatic stump, other parameters are useful to predict failure of pancreatic anastomosis: male sex, high BMI, and prolonged surgery duration were correlated in our series to fistula occurrence. Furthermore, multiparametric scores, including morphological, clinical, and biochemical parameters, could be useful in prediction of pancreatic anastomosis failure; their value should be analyzed and validated in further studies.

## 5. Conclusions

The identification of factors influencing the failure of pancreatic anastomosis is useful for patients management (drainage, type of reconstruction, radiological evaluation, and postoperative care) allowing for their stratification in high or low risk.

Pancreatic texture, assessed by the surgeon, is a significant determining factor for pancreatic fistula and high grade pancreatic fistula and corresponds to pancreatic fibrosis grade.

Moreover, careful consideration should be given to the larger pancreatic stumps, small Wirsung duct, wide pancreatic remnant mobilization, and the duct decentralization on the stump anteroposterior axis. These morphological features influence anastomosis failure.

Our study confirmed that a standardized intraoperative assessment of pancreatic anatomical features of the pancreatic stump by experienced pancreatic surgeon can predict different levels of risk for the development of postoperative pancreatic fistula.

## Figures and Tables

**Table 1:** Fibrosis and inflammation grading at histology and final score computation.

Pancreatic stump fibrosis-and-inflammation score			
Grade of fibrosis	Score	Grade of inflammation	Score
No fibrosis	0	No inflammation	0
Local Fibrosis (lobular or acinar)	1	Focal inflammation	1
Lobular generalized fibrosis	2	Generalized inflammation	2
Acinar generalized fibrosis	3		
Subtotal fibrosis	4		

Final score computation			

0–2	Regular pancreas or mild alterations: perilobular or periacinar fibrosis, no inflammation or focal inflammation		
3-4	Moderate tissue alterations: perilobular generalized fibrosis, focal or diffuse inflammatory infiltrate		
5-6	Generalized fibrosis with total or subtotal disruption of acinar structure, intense inflammation		

**Table 2:** Descriptive statistics regarding the study population.

Patients population (*n* = 145)	
Age (yy)	65,97 ± 10,74
Sex (M : F)	85 : 60
BMI (kg/m^2^)	23,94 ± 4,08
ASA score	
1	18 (12,4%)
2	83 (57,2%)
3	41 (28,3%)
4	3 (2,1%)
Diabetes	32 (22,1%)
Preoperative biliary stenting	76 (52,4%)
Albumin (g/dL)	4,12 ± 0,37
Cholinesterase (kUI/L)	8,41 ± 2,31
Total blood protein (g/L)	68,65 ± 6,15
Hemoglobin (g/dL)	12,99 ± 1,60
Hematocrit (%)	38,29 ± 4,56
Neoadjuvant CT-RT	12 (8,30%)
Intraoperative blood loss (mL)	373,10 ± 265,75
Surgery time (min)	468,91 ± 68,61
Blood transfusion	26 (18%)
Pylorus preserving PD	128 (88,2%)
LOS (days, median, and range)	12 (3–108)
Overall morbidity	89 (59,3%)
Mortality	6 (4,1%)
Clavien	
0	59 (40,70%)
I	6 (4,1%)
II	48 (33,1%)
IIIa	16 (11,0%)
IIIb	7 (4,8%)
IV	3 (2,10%)
V	6 (4,10%)
POPF	41 (28,3%)
Grade A	18 (12,4%)
Grade B	16 (11,0%)
Grade C	7 (4,8%)
Biliary fistula	10 (6,90%)
Lymphatic fistula	9 (6,20%)
Postoperative bleeding	19 (13,10%)
Delayed gastric empting	4 (2,8%)
Reintervention	9 (6,20%)
Readmission	9 (6,20%)

**Table 3:** Univariate analysis of preoperative and intraoperative clinical risk factors for POPF.

	POPF (*n* = 41)	No POPF (*n* = 104)	*P* value
Age	63,99 ± 11,39	66,75 ± 10,42	0,164
Sex (M : F)	31 : 10	54 : 50	0,009
BMI	25,57 ± 3,50	23,29 ± 4,12	0,002
ASA score			
1	8 (19,5%)	10 (9,6%)	0,228
2	24 (58,5%)	59 (56,7%)	
3	9 (22%)	32 (30,8%)	
4	0	3 (2,9%)	
Diabetes	5 (12,2%)	27 (26%)	0,072
Jaundice at surgery	13 (34,6%)	36 (33%)	0,886
Preoperative biliary stenting	22 (55%)	54 (52,4%)	0,782
Albumin	4,16 ± 0,34	4,10 ± 0,38	0,421
Cholinesterase	8,29 ± 1,91	8,46 ± 2,46	0,699
Total blood protein	69,77 ± 5,11	68,22 ± 6,48	0,177
Hemoglobin	13,39 ± 1,66	12,84 ± 1,56	0,073
Bile infection	26 (58,3%)	60 (65%)	0,459
Blood loss (mL)	419 ± 264	354 ± 265	0,188
Surgery time (min)	498,88 ± 57,41	456,98 ± 69,28	0,001
Blood transfusion	6 (14,6%)	20 (19,2%)	0,516
Gastric resection	5 (12,2%)	12 (11,5%)	0,912

**Table 4:** Univariate analysis of morphological features of pancreatic stump as risk factors for POPF.

	Patients with POPF (*n* = 41)	Patients without POPF (*n* = 104)	*P* value	Patients with grade B-C POPF (*n* = 23)	Other patients (*n* = 122)	*P* value
Pancreatic texture by surgeon						
Soft (*n* = 45)	30	15	<0,001	19	26	<0,001
Medium or hard (*n* = 100)	11	89		4	96	
Wirsung diameter (mm)	3,19 ± 1,21	4,29 ± 1,73	<0,001	3,08 ± 1,42	4,14 ± 1,69	0,006
≤3 mm (*n* = 63)	26	37	0,002	15	48	0,022
>3 mm (*n* = 82)	15	67		8	74	
Wirsung decentralization						
Anteroposterior axis	0,31 ± 0,34	0,23 ± 0,30	0,031	0,28 ± 0,40	0,25 ± 0,31	0,131
Craniocaudal axis	−0,17 ± 0,16	−0,12 ± 0,23	0,426	−0,20 ± 0,16	−0,13 ± 0,21	0,412
Stump area (mm^2^)	219,21 ± 113,79	138,23 ± 99,08	<0,001	223,82 ± 110,42	149,59 ± 105,97	0,003
Stump mobilization (mm)	24,26 ± 5,42	20,59 ± 4,02	<0,001	24,34 ± 5,89	21,12 ± 4,33	0,003

**Table 5:** Multivariate logistic regression analysis regarding POPF occurrence.

	*β*	*P* value	CI (inf.–sup.)
Sex	−,142	0,043	−0,259–−0,004
BMI	,129	0,085	−0,002–0,031
Surgery duration	,013	0,166	−0,001–0,001
Stump soft texture	,443	0,000	0,285–0,590
Stump mobilization	,235	0,001	0,093–0,359
Stump area	,020	0,800	−0,001–0,001
Wirsung diameter	−,074	0,340	−0,060–0,001
Wirsung AP decentralization	,010	0,881	−0,176–0,205
Constant		0,044	

**Table 6:** Fibrosis-and-inflammation score and surgeon judgment about pancreatic texture.

Fibrosis-and-inflammation score and surgeon judgment about pancreatic texture				
Final score	Hard texture	Medium texture	Soft texture	*P* value
0–2	3	16	35	<0,001
3-4	12	10	0	
5-6	25	12	0	

**Table 7:** Association between fibrosis-and-inflammation final score and POPF occurrence.

Fibrosis-and-inflammation score and POPF development			
Final score	POPF (*n* = 30)	No POPF (*n* = 83)	*P* value
0–2	27	26	<0,001
3-4	3	19	
5-6	0	38	
